# Genomic insights into *Candidatus* Argiopiplasma dusa, a bacterial symbiont of the wasp spider *Argiope bruennichi*

**DOI:** 10.1093/femsle/fnag103

**Published:** 2026-09-22

**Authors:** Larissa M Busch, Monica M Sheffer, Nina Dombrowski, Henrik Krehenwinkel, Stefan Prost, Anja Spang, Gabriele Uhl, Tim Urich, Katharina J Hoff, Mia M Bengtsson

**Affiliations:** Center for Functional Genomics of Microbes, University of Greifswald, 17489 Greifswald, Germany; Institute of Mathematics and Computer Science, University of Greifswald, 17489Greifswald, Germany; Institute of Microbiology, University of Greifswald, 17489 Greifswald, Germany; Department of Functional Genomics, Interfaculty Institute for Genetics and Functional Genomics, University Medicine Greifswald, 17489 Greifswald, Germany; Zoological Institute and Museum, University of Greifswald, 17489 Greifswald, Germany; Department of Biological Sciences, California Polytechnic University, Humboldt, Arcata, CA 95521, United States; Department of Marine Microbiology and Biogeochemistry, Royal Netherlands Institute for Sea Research (NIOZ), NL-1790 AB Den Burg, The Netherlands; Department of Evolutionary & Population Biology, Institute for Biodiversity and Ecosystem Dynamics (IBED), University of Amsterdam, 1090 GE Amsterdam, The Netherlands; Department of Biogeography, University of Trier, 54296 Trier, Germany; Ecology and Genetics Research Unit, University of Oulu, 90014 Oulu, Finland; Central Research Laboratories, Natural History Museum Vienna, 1010 Vienna, Austria; Department of Marine Microbiology and Biogeochemistry, Royal Netherlands Institute for Sea Research (NIOZ), NL-1790 AB Den Burg, The Netherlands; Department of Evolutionary & Population Biology, Institute for Biodiversity and Ecosystem Dynamics (IBED), University of Amsterdam, 1090 GE Amsterdam, The Netherlands; Zoological Institute and Museum, University of Greifswald, 17489 Greifswald, Germany; Center for Functional Genomics of Microbes, University of Greifswald, 17489 Greifswald, Germany; Institute of Microbiology, University of Greifswald, 17489 Greifswald, Germany; Center for Functional Genomics of Microbes, University of Greifswald, 17489 Greifswald, Germany; Institute of Mathematics and Computer Science, University of Greifswald, 17489Greifswald, Germany; Center for Functional Genomics of Microbes, University of Greifswald, 17489 Greifswald, Germany; Institute of Microbiology, University of Greifswald, 17489 Greifswald, Germany

**Keywords:** *Argiope bruennichi*, spider symbiont, symbiosis, *Mycoplasmatota*, *Mycoplasma*, *Candidatus* Argiopiplasma dusa

## Abstract

Bacterial symbionts were shown to have diverse but significant impacts on the fitness of their host in insects, but little is known on the symbionts of spiders and their interactions with their hosts. Here, we assembled and investigated the circular 575 kb genome of *Candidatus* Argiopiplasma dusa, a novel bacterial symbiont of the wasp spider *Argiope bruennichi*. Phylogenomic analysis placed this species within the phylum *Mycoplasmatota*, in a poorly characterized clade that may represent a new order-level lineage or affiliate with the *Mycoplasmatales* order. With 559 predicted genes, the genome is relatively small compared to other *Mycoplasmatota* genomes (on average 969 genes) and has a low GC content of ∼24%. While the genome encodes genes for proteins involved in glycolysis and fermentative acetate production, it revealed minimal biosynthetic capabilities with pathways for nucleotide, amino acid, and vitamin biosynthesis being absent in *Ca*. Argiopiplasma dusa. This suggests a metabolic dependence and an endosymbiotic lifestyle within the spider host. The symbiont was detected in *A. bruennichi* populations across the distribution range of the spider but appears to be absent in certain populations.

## Teaser

The highly reduced genome of the wasp spider's bacterial symbiont suggests an endosymbiotic lifestyle characterized by metabolic dependencies.

## Introduction

Host-adapted bacteria influence the biology of their hosts in many ways, as reproductive parasites, commensals or mutualists (Kaltenpoth et al. 2025). Approximately one third of all sequenced bacterial genomes are from those that live in close interactions with eukaryotic hosts (Toft and Andersson 2010). Depending on their level of host dependency and the intimacy of their interactions, their genomic characteristics differ widely. For instance, obligate intracellular bacterial insect symbionts are often characterized by extremely reduced genomes (Moran and Bennett 2014) that have evolved independently in multiple bacterial clades from free-living bacterial ancestors with larger genomes (Rogers et al. 1985, Ochman 2005, Moran and Bennett 2014). The loss of genes in symbionts can be explained by their increasing reliance on gene products of their host organism and a stable host-associated environment with less essential reliance on stress response systems.

Although endosymbiosis is well-documented in a number of insect taxa and especially well-explored in those groups that exploit challenging nutritional resources such as leafhoppers, cicadas, aphids, and some beetles (Bennett et al. 2024, Kaltenpoth et al. 2025), the currently known host taxa do not reflect the diversity of potential arthropod hosts, thereby limiting our understanding of the range of arthropod-bacterial symbiosis, their functional and evolutionary roles, as well as their genomic features (Mathieson et al. 2025).

In particular, spiders are among the less-studied arthropod groups. However, reproductive parasites such as *Wolbachia, Rickettsia, Cardinium*, and *Spiroplasma* and additional symbiont species have been discovered in several spider species (Vanthournout and Hendrickx 2015, Zhang et al. 2017, 2018, Sheffer et al. 2020, White et al. 2020, Perez-Lamarque et al. 2022, Nariman et al. 2025). Spiders have an obligate predatory lifestyle (Nentwig 1987), which implies potential transmission of symbionts into spiders from their prey (Kennedy et al. 2020), and which might be associated with a specialized nutritional roles of symbionts within their spider hosts, for example linked to decomposition of abundant prey constituents.

A genomic study of two populations (Germany, Estonia) of the wasp spider *Argiope bruennichi* revealed the presence of a previously unknown bacterial endosymbiont (Sheffer et al. 2020, 2021) referred to as DUSA (dominant unknown symbiont of *Argiope*). Here, we refer to this symbiont as *Candidatus* Argiopiplasma dusa (see description), where “dusa” is an arbitrary combination of letters to be treated as a noun in apposition (fem.). The fact that the symbiont was found in multiple tissues (leg, prosoma, hemolymph, book lungs, ovaries, silk glands, midgut, and fecal pellets; Sheffer et al. 2020) demonstrates that it is not part of the microbiome of the digestive tract but likely represents a systemic endosymbiont, possibly with an intracellular lifestyle. DUSA was shown to be affiliated with the *Mollicutes*, the major class in the phylum *Mycoplasmatota*, though the 16S rRNA gene had a remarkably low identity (<85%) to 16S rRNA genes in public databases (Sheffer et al. 2020).

Currently known representatives of *Mollicutes* are small micro-organisms (<1 µm in diameter) that lack a cell wall (Gasparich 2010, Razin and Hayflick 2010). The major genera of the *Mollicutes, Mycoplasma*, and *Spiroplasma*, are widespread and comprise mutualists and parasites of many animals and plants (Razin et al. 1998, Yavlovich et al. 2004, Gasparich 2010, Razin and Hayflick 2010, Zhang et al. 2018, White et al. 2020).

Here, we assembled the full circular genome of *Ca*. A. dusa from metagenome datasets, that were originally used to construct the genome of the host spider *A. bruennichi* (Sheffer et al. 2021) and to describe its range expansion (Krehenwinkel et al. 2015). Our genomic and phylogenetic analyses reveal its metabolic potential and phylogenetic placement within the *Mycoplasmatota*. Furthermore, by investigating metagenomes of geographically separated European and Japanese spider host populations, we were able to shed light into the biogeography of *Ca*. Argiopiplasma dusa and its population dynamics.

## Materials and methods

### Genome assembly and annotation

We used previously published (meta)genome sequencing reads that were originally produced for assembling the wasp spider genome (Sheffer et al. 2021), but also contained endosymbiont genome reads, to generate the *Ca*. Argiopiplasma dusa symbiont genome assembly. We assessed three different assembly strategies to separate *A. bruennichi* genome information, *Ca*. A. dusa genome information and contaminant/off-target genetic information (Fig. S1). The strategies and assembly methods are described in detail in the Supplementary method S1. In brief, we used PacBio whole genome reads (Sequence Read Archive: SRR11652932; (Sheffer et al. 2021)) of a female *A. bruennichi* from Portugal to generate the baseline genome assembly, and Illumina paired-end reads (European Nucleotide Archive: ERR574428; (Krehenwinkel et al. 2015)) of several populations for assembly polishing. PacBio reads were trimmed after removing duplicated reads by SeqKit rmdup (v0.12.0; (Shen et al. 2016)) using Filtlong (v0.2.0; settings:–min_length 1000 –keep_percent 90; github.com/rrwick/Filtlong) and Illumina reads were trimmed using Trimmomatic (v0.39; settings: ILLUMINACLIP:TruSeq3-PE.fa:2:30:10:2:keepBothReads LEADING:3 TRAILING:3 MINLEN:36; (Bolger et al. 2014)).

To create the final selected assembly, we binned the PacBio reads using BlobTools (v1.1.1; (Laetsch and Blaxter 2017)) and mapping of hits to the UniProt database (release: 2019_02; (The UniProt Consortium 2019)) by DIAMOND (v0.9.35.136; (Buchfink et al. 2015)). Reads taxonomically classified as “Tenericutes” were selected. These reads were used as the starting assembly for a “map-and-assemble” approach (Wang and Chandler 2016): all reads were iteratively mapped to the assembly using Minimap2 (v2.17; settings: -ax map-pb; (Li 2018)) and all mapped reads were re-assembled with Flye (v2.7; settings: –genome-size 1 m; (Kolmogorov et al. 2019)) until there was no increase of assembly genome size. The resulting assembly was used as a draft assembly, after which all mapping reads were re-assembled using Raven (v1.1.10; (Vaser and Šikić 2019)) and polished twice with Racon (v1.4.15; (Vaser et al. 2017)) using the PacBio reads against the assembly by Minimap2 and five times with Pilon (v1.23; (Walker et al. 2014)) using the Illumina reads mapped against the assembly by BWA-MEM (v0.7.17; (Li 2013)) and sorted with SAMtools (v1.10; (Li et al. 2009)). The polished assembly was checked for contaminants using BlobTools, for circularity using Bandage (v0.8.1; (Wick et al. 2015)) and assembly metrics were computed with QUAST (v5.0.2; (Gurevich et al. 2013)). Additionally, the 16S rRNA gene in the assembly was identified and compared to that of the symbiont identified in the previous *A. bruennichi* microbiome study (Sheffer et al. 2020) using NCBI-Blast+ (v2.10.1+; (Camacho et al. 2009)). The assembly was submitted under accession CP070274, BioProject: PRJNA629526 and BioSample: SAMN17496637 at the NCBI. Based on the PGAP (Tatusova et al. 2016) structural gene annotation obtained from the RefSeq pipeline, completeness of the genome assembly was estimated with BUSCO (v4.1.1;(Seppey et al. 2019)) using the *Mycoplasmatota* OrthoDB v10 (Kriventseva et al. 2019) reference database.

### Phylogenetic analysis

Maximum likelihood phylogenetic reconstructions were performed using 26 single-copy markers from a previously established marker gene set and a backbone of 1205 bacterial genomes (Moody et al. 2022). To identify marker protein homologs, amino acid sequences (proteins) from all genomes of interest were combined and used in an hmmsearch (v3.1b2, settings: hmmsearch –tblout output–domtblout –notextw; (Finn et al. 2011)) against the COG profiles database (Tatusov et al. 2003). Afterwards, the 26 marker proteins were extracted and if more than one hit was assigned to each marker for a single genome, only the best hit was retained based on the best bit-score and e-value. Marker proteins were individually aligned using MAFFT L-INS-i (v7.407, settings: –anysymbol –reorder; (Katoh 2002)) and trimmed using BMGE (v1.12, settings: -t AA -m BLOSUM30 -b 2 -h 0.55; (Criscuolo and Gribaldo 2010)). The trimmed amino acid alignments were concatenated using catfasta2phyml (github.com/nylander/catfasta2phyml) and a phylogenetic tree was generated using IQ-TREE (v2.1.2; settings: -m LG + F + R -bb 1000 -alrt 1000; (Minh et al. 2020)). This tree was used to down sample the taxon selection to 479 bacterial genomes (Table S1; Fig. S1). From this taxon set, the 26 marker proteins were extracted, aligned and trimmed using MAFFT L-INS-i and BMGE as described above. Alignments were concatenated and a phylogenetic tree was generated using IQ-TREE (settings: -m LG + C60 + F + R -bb 1000 -alrt 1000).

SlowFaster (v1; (Kostka et al. 2008)) was used with default settings on the concatenated alignment to remove fast-evolving sites. Additionally, heterogeneous sites were removed using alignment_pruner.pl (settings: –chi2_prune, github.com/novigit/davinciCode/blob/master/perl). For both approaches, sites were removed in a step-wise manner removing 10%, 20%, 30%, and 40% of sites (Figs. S2-9). Afterwards, phylogenetic trees were generated using IQ-TREE (settings: -m LG + C20 + F + R -bb 1000 -alrt 1000).

For further functional annotation, we analyzed the *Ca*. A. dusa symbiont genome and 478 bacterial genomes. Rapid gene calling was performed using Prokka (Seemann 2014), with genetic code 11 applied to all genomes except Mycoplasma, for which genetic code 4 was used. Afterwards, the generated proteins were compared against several databases (Supplementary methods S2).

### Population genetic analysis

SNP analysis to investigate the population structure of *Ca*. A. dusa was conducted using Illumina reads obtained from *A. bruennichi* populations in Japan (five specimen data sets), Sweden, the Baltic region, Portugal, Italy (European Nucleotide Archive: ERR574428; (Krehenwinkel et al. 2015), the Azores and Madeira. The population specific reads were mapped against the *Ca*. A. dusa genome by BWA-MEM (v0.7.17; (Li 2013)). SNP calling was then performed using SAMtools and BCFtools (v1.10; (Danecek et al. 2021)) assuming a ploidy of 1. The top 90% quality sites identified in all populations were analyzed by principal component analysis (PCA) and hierarchical clustering in R (v4.1.2) using the packages tidyverse (v2.0.0; (Wickham et al. 2019)), FactoMineR (v2.8; (Lê et al. 2008)), and factoextra (v1.0.7) to characterize the *Ca*. A. dusa population differences according to the variant calls.

## Results

### *Ca*. Argiopiplasma dusa genome characteristics suggesting genome reduction

The genome assembly of *Ca*. A. dusa (accession CP070274) using a metagenomics approach (Supplementary Methods S1), resulted in a set of 18 candidate assemblies. We selected the assembly with the largest genome size, 575 551 bp, present in one circular contig as representative assembly for the genome of *Ca*. A. dusa genome (Table 1). The single 16S rRNA gene detected on this contig is 99.8% identical to the previously reported full-length 16S rRNA gene sequence of *Ca*. A. dusa in the *A. bruennichi* microbiome (Sheffer et al. 2020). The genome contains 526 protein coding sequences (CDS) in addition to 27 tRNA genes and one of each for 5S, 16S,and 23S rRNA gene, respectively, as well as three ncRNAs.

**Table 1:** Assembly statistics. Assembly metrics were computed with QUAST v5.0.2 (Gurevich et al. 2013) and DUSA 16S rRNA (Sheffer et al.(Sheffer et al. 2020)) gene sequence identity was analyzed with Blast+ (Camacho et al. 2009). CDS Annotation done with NCBI Prokaryotic Genome Annotation Pipeline (PGAP) (Tatusova et al. 2016).

Assembly statistics	
# contigs	1
Assembly size [bp]	575 551
N50 [bp]	575 551
GC (%)	24.12
16S rRNA gene identity	99.8%
# genes	559
Complete BUSCOs	82.7%
Fragmented BUSCOs	1.3%
Missing BUSCOs	16.0%

The previous analysis of the 16S rRNA gene sequence placed the endosymbiont into the clade *Mycoplasmatota* (former *Tenericutes*) (Sheffer et al. 2020). Comparison with 102 *Mycoplasmatota* genomes in the RefSeq database, which range from 0.56–1.97 Mb (mean: 1.03) and encode for 560–4727 genes (mean: 969), revealed that the genome of *Ca*. A. dusa at 0.58 Mb was among the smallest *Mycoplasmatota* characterized to date. This could reflect a reduced genomic complexity often observed for endosymbionts (Román-Escrivá et al. 2025). In agreement with the previous observation that many endosymbiont genomes have an AT bias with a consequently low GC content (McCutcheon et al. 2009, Román-Escrivá et al. 2025), the *Ca*. A. dusa genome had a low GC content (24.1%), even lower than that of most *Mycoplasmatota* reference genomes (range 23.1%–40.0%; mean 27.9%) and also lower than the GC content of the *A. bruennichi* genome (29.3%; (Sheffer et al. 2021)). *Ca*. A. dusa encoded 82.7% (n = 124/150 genes) of the complete *Mycoplasmatota* OrthoDB v10 marker genes (Kriventseva et al. 2019), while 1.3% of genes (n = 2) were fragmented and 16% were missing (n = 24). None of the 24 missing *Mycoplasmatota* marker genes (Supplementary Data Table S2) were identified in any of the alternative genome assemblies, indicating that these genes are likely missing in the *Ca*. A. dusa genome.

### *Ca*. A. dusa falls into a deep-branching *Mycoplasmatota* clade

**Figure 1:**
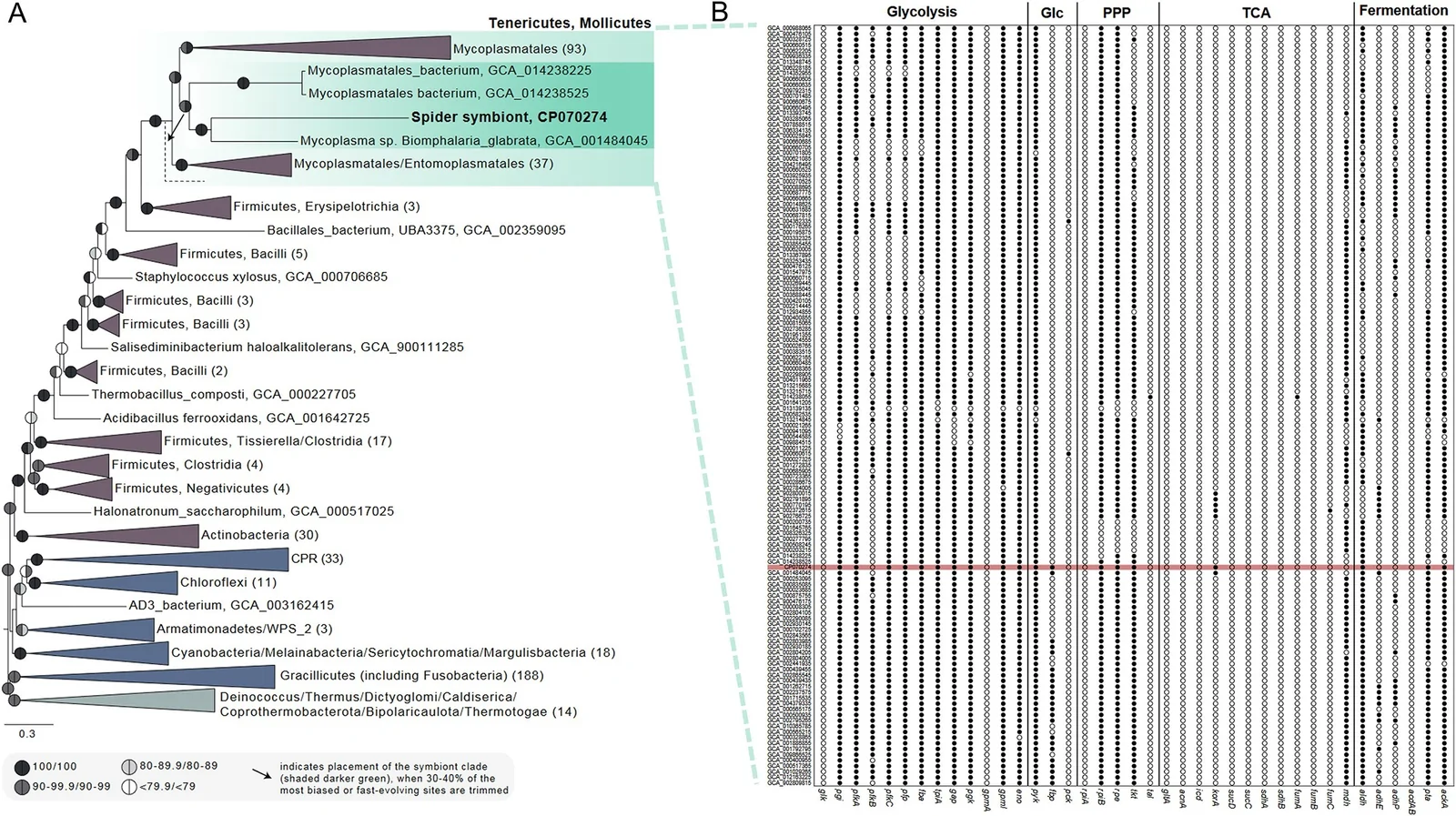
Phylogenetic placement of *Ca*. A. dusa(A). Comparison of predicted C-metabolism of *Mycoplasmatales* (see Supplementary Figure 12) (B). Gene content of *Ca*. A. dusa is highlighted in red. Arrow points to the alternative position obtained when removing 30%–40% of the most compositionally or fast-evolving biased sites (see Supplementary Fig. 1 and 2).

Phylogenetic analyses of *Ca*. A. dusa using 26 marker genes revealed its putative placement within the class *Mollicutes* in the phylum *Mycoplasmatota*. Together with three other taxa, *Ca*. A. dusa may either be affiliated with the *Mycoplasmatales* order or form a separate order-level lineage sister to *Mycoplasmatales* or *Mycoplasmatales* and *Entomoplasmatales* clades combined (Fig. 1 and S1-9). Specifically, while the full alignment recovered a placement between *Mycoplasmatales* and *Mycoplasmatales/Entomoplasmatales*, upon filtering of the 30%–40% most compositionally or fast-evolving sites, this clade emerged basal to all *Mycoplasmatales* and *Entomoplasmatales* (Fig. 1A). The closest sequenced relatives to *Ca* A. dusa are characterized by similarly small genome sizes, and were found in a snail (host: *Biomphalaria glabrata*, symbiont genome size: 0.65 Mb, accession number: GCA_001484045; (Adema et al. 2017)) and a glass sponge collected from the Scotia Shelf (host: *Vazella pourtalesii*, symbiont genome size: 0.67 Mb & 0.68 Mb,GCA_014238525 & GCA_014238225; (Bayer et al. 2020)).

The long branches separating those *Mycoplasmatota* from each other and their next relatives, may indicate that these organisms have experienced relatively fast evolutionary rates due to their symbiotic lifestyle as commonly observed in recent insect symbionts (O’Fallon 2008, Bennett et al. 2014, Gerth et al. 2021, Wang 2026 ). Conversely, the phylogenetic diversity of putative hosts indicates that this *Mycoplasmatota* clade is likely currently under-sampled and may comprise additional members infecting a broad range of eukaryotic host families.

### Functional gene content and metabolic potential

**Figure 2:**
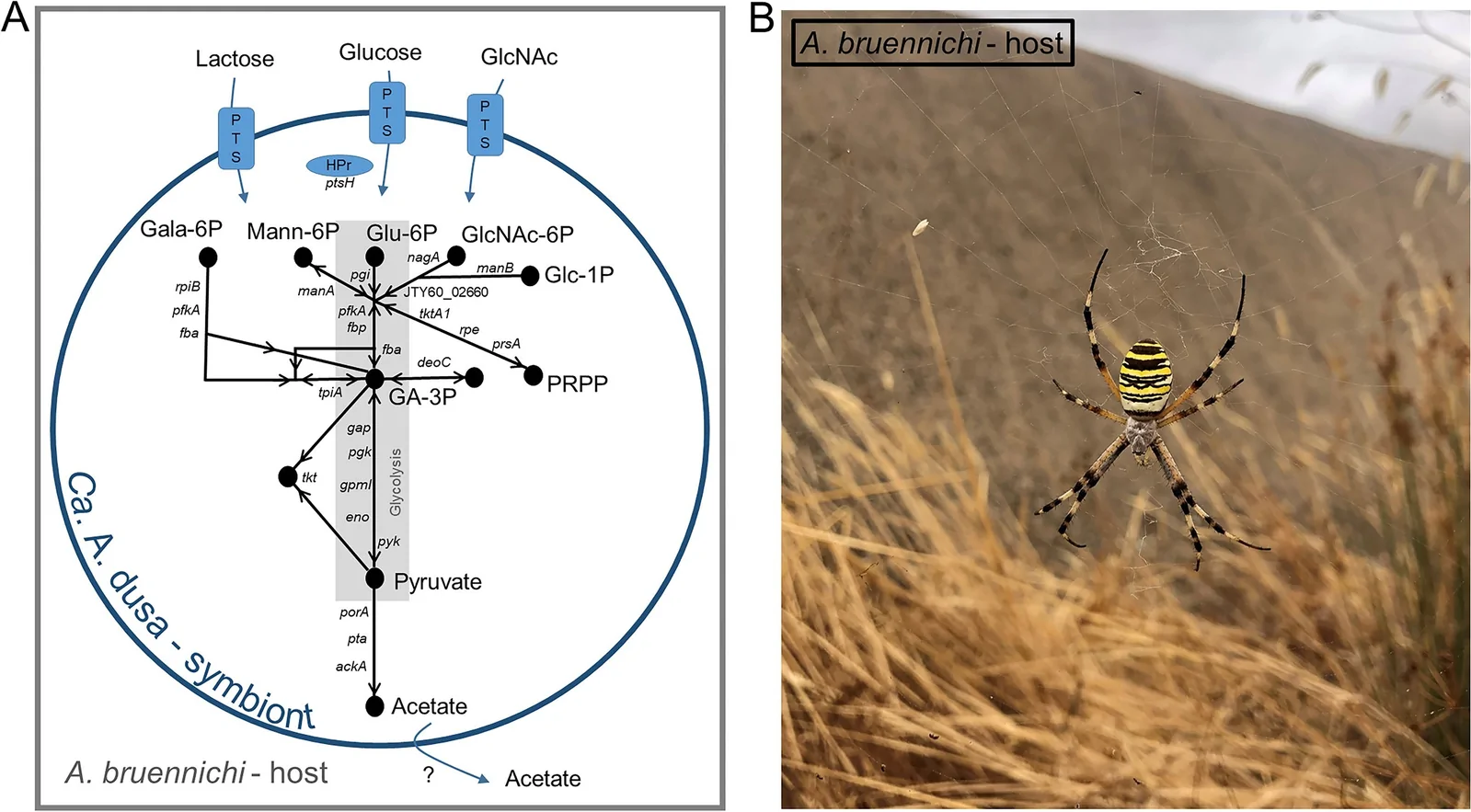
Model of the central C-metabolism of *Ca*. A. dusa. (A) Based on the functional annotation of *Ca*. A. dusa genes, the metabolic potential is depicted. Sugars are likely provided by the *A. bruennichi* host, whereas the fermentative product acetate may be provided by the symbiont and further metabolized by the host. PTS: Phosphotransferasesystem. HPr: Histidine-containing phosphocarrier protein. Glu: Glucose. GlcNAc: N-Acetylglucosamine. Gala-6P: Galactose-6-phosphate. Mann-6P: Mannose-6-phosphate. Glu-6P: Glucose-6-phosphate. GlcNAc-6P: N-Acetylglucosamine-6-phosphate. GlcN-1P: Glucosamine-1-phosphate. PRPP: Phosphoribosyl pyrophosphate. GA-3P: Glyceraldehyde-3P. Main metabolites are indicated as filled circles. A detailed annotation is given in Fig. S2. (B) Photo of the host organism *A. bruennichi* taken in 2019 of a specimen in Portugal.

Similar to other *Mycoplasmatota* genomes (Pollack et al. 1997) with the exception of the highly reduced *Oceanoplasmataceae* and *Moeniiplasmataceae* (Vohsen et al. 2024) as well as some *Phytoplasma* species (Kube et al. 2012), the genome of *Ca*. A. dusa encodes genes for complete glycolysis pathway, while lacking genes for the oxidative citric acid cycle (Fig. 2A and Fig S11, Table S3). Instead, acetyl-CoA, produced from pyruvate oxidoreductase (*porA*) after glycolysis, is likely transformed to acetate in a short fermentative pathway *via* phosphate acetyltransferase (*pta*) and acetate kinase (*ackA*, Fig. 2) yielding ATP *via* substrate level phosphorylation. Of note, the capability to ferment sugars varies across the *Mollicutes* species (Miles 1992), thus this metabolic property might play a role in establishing the symbiosis of *A. bruennichi* and *Ca*. A. dusa. No genes encoding proteins of aerobic or anaerobic respiratory chains were detected. Diverse simple carbohydrates, such as fructose, glucose, mannose, galactose, N-acetyl-glucosamine are predicted to be fueled into the glycolysis pathway based on the detected PTS system components and downstream enzymes (*pgi, pfkA, pfkC, ptp, fba, tpiA, gap, pgk, gpmI, eno, pyk*) (Figs. 1B and 2A, Table S3). Differing from most *Mycoplasmatota* species, *Ca*. A. dusa also encodes genes involved in gluconeogenesis (*fbp*; Fig. 1B). Given this, *Ca*. A. dusa might provide acetate and/or monosaccharides to its spider host. No genes encoding enzymes of fatty acid biosynthesis are found, which is in line with the dependence of multiple *Mycoplasma* species on exogenous fatty acids and sterols (Kornspan and Rottem 2012); however, the genes encoding enzymes of the terminal steps of phospholipid synthesis are identified (*plsC, pgsA, Cls*), indicating a dependency on host fatty acids commonly observed in genome reduced endosymbionts (McCutcheon et al. 2024). The small *Ca*. A. dusa genome encodes very few enzymes for amino acid biosynthesis, again indicating metabolic dependency on the spider host. However, the exchange of metabolites between the host and the endosymbionts remains to be studied experimentally in detail. While the nucleic acid metabolism is predicted to be similar to other *Mycoplasmatota*, we did not find any alternative vitamin biosynthesis pathways (Table S3).

### *Ca*. A. dusa populations differ in regard to their geographic distribution

The thermophilic wasp spider *A. bruennichi* (Fig. 2B) has a broad global distribution and is currently undergoing a natural poleward range expansion partly caused by climate change (Krehenwinkel and Tautz 2013, Krehenwinkel and Pekar 2015, Krehenwinkel et al. 2015, 2016, Wawer et al. 2017, Wawer and Wytwer 2020, Wolz et al. 2020, Sheffer et al. 2026). Therefore, we asked if all populations carry the *Ca*. A. dusa symbiont and if there are geographic differences in the *Ca*. A. dusa populations as the carriage of *Ca*. A. dusa has only been demonstrated for German and Estonian *A. bruennichi* populations (Sheffer et al. 2020). To shed light on the presence and genome variation of *Ca*. A. dusa in different populations of the spider host, we performed single nucleotide variant (SNV) analysis of seven geographically distinct populations. Mapping of Illumina reads from such distinct *A. bruennichi* populations to the *Ca*. A. dusa genome revealed a separation of *Ca*. A. dusa genomes according to their geographic origin. Of note, not all investigated populations harbored the symbiont; specifically, it appears to be absent from three of the five analyzed Japanese populations (Table S4).

**Figure 3:**
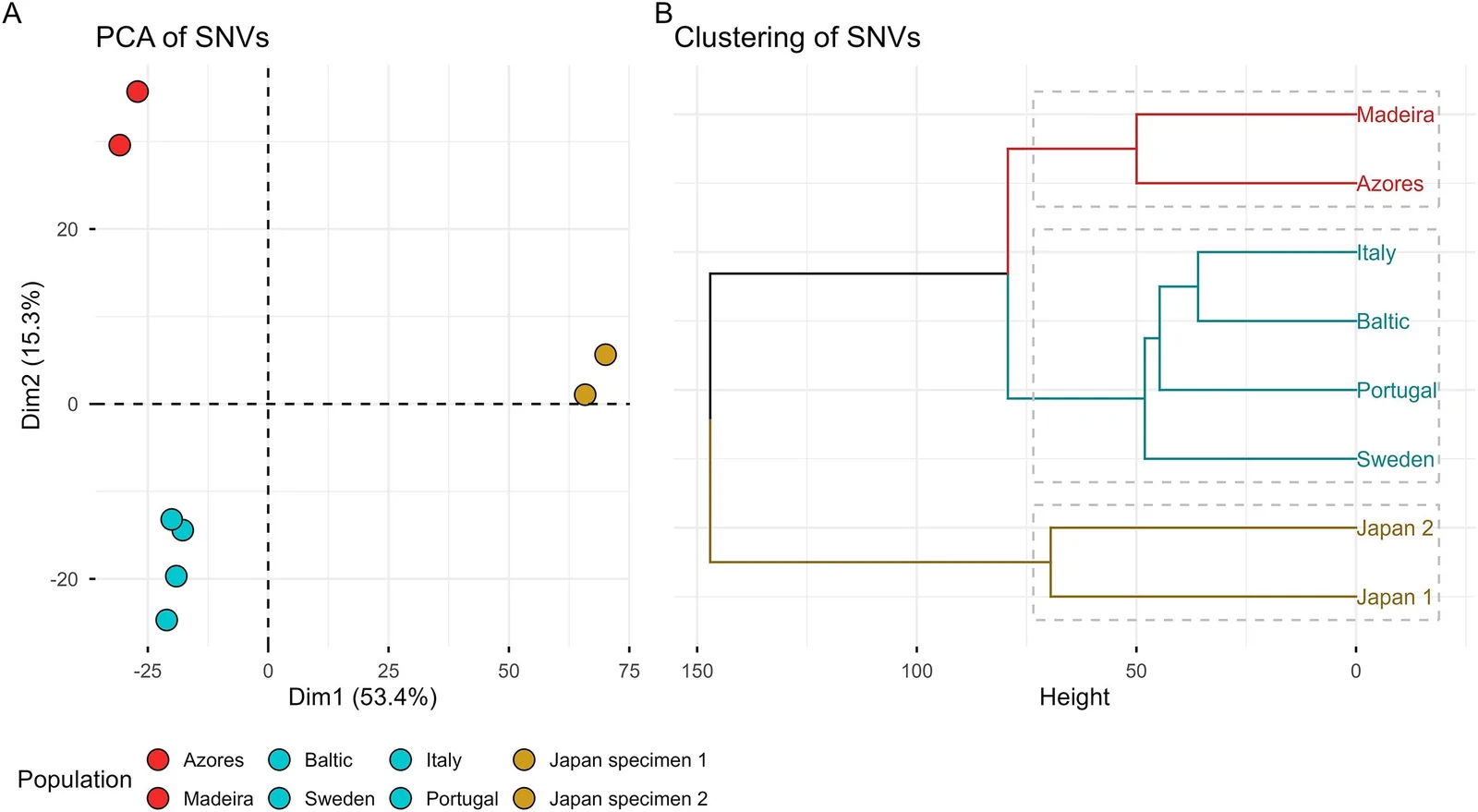
Genome SNV analysis of *Ca*. A. dusa from different populations of *A. bruennichi*. Illumina reads generated for *A. bruennichi* populations/specimens from Japan, Europe (Baltic region, Sweden, Italy, Portugal), and Atlantic islands (Azores, Madeira) were mapped to the *Ca*. A. dusa genome. Corresponding *Ca*. A. dusa population SNV calls were visualized as principal component analysis(A) and hierarchically clustered (B).

Principal component analysis (PCA) and hierarchical clustering of Japanese, European and Atlantic Ocean populations according to the SNV calls present in all analyzed populations shows that the geographic distribution is reflected by the genetic variance of *Ca*. A. dusa (Fig. 3A and B) suggesting different *Ca*. A. dusa lineages. The divergence of microbial genomes also reflects the Palearctic-wide phylogeographic divergence pattern of the host spider (Krehenwinkel et al. 2016), which further supports a shared demographic history of the host and the endosymbiont.

## Discussion

A proposed symbiont of *Mycoplasmatota* clade was first detected in *A. bruennichi* spider populations from Germany and Estonia *via* 16S rRNA gene amplicon sequencing (Sheffer et al. 2020). To study this symbiont in detail, we assembled and annotated the whole genome of this organism using a metagenomics approach from shotgun sequencing data of whole *A. bruennichi* tissues (Sheffer et al. 2021). This approach highlights the potential of reanalysis of available metagenomics data to discover bacteria that occur in natural populations of diverse hosts and provide first insights into the possible role they may play in their host biology and evolutionary history.

Our current assembly and phylogenetic analyses of the symbiont demonstrates that this organism is only distantly related to other known symbionts of the orders *Mycoplasmatales* and *Entomoplasmatales* within the *Mollicutes* class in the phylum *Mycoplasmatota*. The closest currently known related genomes of *Ca*. Argiopiplasma dusa were recovered from such diverse hosts as the freshwater snail *Biomphalaria glabrata* (Adema et al. 2017) and a marine glass sponge *Vazella pourtalesii* (Bayer et al. 2020). That the symbiont bacteria are associated to hosts, which are scattered across the tree of life and occur in very different habitats, may be a consequence of missing observations from other host species and habitats. However, a wider search of the 16S rRNA gene sequence of *Ca*. A. dusa against the NCBI SRA (sequence read archive) did not recover any closely related sequences from other spiders or insects (data not shown) and *Ca*. A. dusa was also not identified in a range of closely related *Argiope* species (H. Krehenwinkel, personal observation).

This raises questions about the origin and dynamics of the symbiotic interaction with the wasp spider. Reanalysis of metagenomics sequencing data of several *A. bruennichi* populations indicated that only two out of five Japanese populations harbor *Ca*. A. dusa, which might suggest that it is not an obligate, primary endosymbiont. The Japanese population is likely the most basal population of the studied spider populations as eastern Asia is a major glacial refugium (Krehenwinkel et al. 2016). Consequently, it remains to be determined whether the symbiosis was ancestral to this spider species with subsequent loss in some populations or was acquired independently in some populations later on. However, reads mapping to the symbiont genome in samples from *A. bruennichi* populations varied across several SNVs, in a pattern consistent with geographic distribution and genetic variation of the spider host (Wawer et al. 2017), indicating a possible co-evolution of the spider host and the *Ca*. A. dusa symbiont. Given the recent geographic range expansion of the host, this suggests that symbiont evolution may be rapid. In line with other arthropod-symbiont systems (Goodacre and Martin 2012), we hypothesize that the symbiont may have a potential role in phenotypic traits linked to geographic expansion, such as dispersal, cold tolerance, or reproduction. Indeed, previous work has shown that evolutionary diversification and adaptation of a wide range of host species is associated with carrying of symbionts. Symbiosis may facilitate harnessing new forms of energy, accession of limiting nutrients and outsourcing of critical defense functions against natural enemies (Cornwallis et al 2023). For insects a clear correlation of carriage of (obligate) symbionts and the rate of diversification within one species is demonstrated (Cornwallis et al. 2023, Bennett et al. 2024). Analysis of the metabolic potential of *Ca*. A. dusa indicates that it may supply acetate and monosaccharides to its spider host. These compounds could offer nutritional benefits and/or function as antifreeze agents for overwintering juvenile *A. bruennichi*, similar to other low molecular weight solutes increasing thermal hysteresis in spiders (e.g. Duman 1979). The recent poleward expansion of *A. bruennichi*, which surpasses the rate of climate change-induced warming (Krehenwinkel et al. 2015), goes in line with adaptations to more severe temperatures, particularly for the spiderlings that overwinter in the egg sac (Ortiz-Movliav et al. 2025, Sheffer et al. 2026). Notably, metabolic differences have been observed between spiderlings from the expansion edge and those from the center of the range (Sheffer et al. 2026). The potential role of *Ca*. A. dusa in facilitating cold adaptation in the host remains to be explored.

Prospective experimental work should focus on testing hypotheses on the potential functional role of *Ca*. A. dusa in its host. Comparative genomics has provided insights into reductive genome evolution and associated loss in functional abilities of obligate endosymbionts (Moya et al. 2008, Moran and Bennett 2014).

Based on the previous discovery of 16S rRNA genes of *Ca*. A. dusa in all host tissues studied (legs, prosoma, ovaries, midgut, silk glands, hemolymph and fecal pellets) as well as in newly hatched spiderlings, indicates that the symbiont is located within the host body and is maternally transmitted possibly *via* eggs or the egg-laying process (Sheffer et al. 2020). This points towards a systemic distribution of the symbiont within the host and a potential for vertical transmission to the offspring. However, we cannot distinguish whether the symbiont is intracellular, or otherwise located within the host tissue or present in the hemolymph. The small genome size and level of genome reduction, however, are consistent with *Ca*. A. dusa potentially being an intracellular symbiont, dependent on its host for basic metabolic functions (Moran and Bennett 2014). Our understanding of the interaction between symbiont and host, including any beneficial or antagonistic effects, is preliminary. The genomic analyses suggest that *Ca*. A. dusa can likely metabolize simple sugars and N-acetyl-glucosamine resulting in acetate as an end product. Given this, *Ca*. A. dusa might provide acetate, likely *via* passive diffusion across the bacterial cell membrane, to its spider host, which could be assimilated into the host’s central carbon metabolism, as shown for other arthropod-bacterial symbioses (Ankrah et al. 2018)—however this is highly speculative and requires further investigations in experimental studies. Metabolic interactions between fermentative bacteria producing short-chain fatty acids, such as acetate, propionate or butyrate and their animal host cells are widespread (McFall-Ngai et al. 2013).

The absence of biosynthetic pathways for vitamins, amino acids or specialized enzymes for digesting typical dietary components of the spider (insect biomass), do not allow us to infer any typical mutualistic interactions between host and symbiont.

Altogether, the evolutionary history and symbiotic function of *Ca*. A. dusa in the spider *A. bruennichi* remains enigmatic. We encourage future studies to widen the search for *Ca*. A. dusa and related organisms in other potential hosts to explore the role of *Ca*. A. dusa in symbiosis. A logical next step to investigate the specific role for *A. bruennichi* would be to explore whether the symbiont is intercellular or extracellular in the tissues of *A. bruennichi* and to carry out experimental studies on populations with and without symbiont to shed further light onto the functional characteristics of the symbiosis between *A. bruennichi* and *Ca.* A. dusa.

### Description of *Candidatus* Argiopiplasma dusa

We generated the symbiont genome assembly based on whole genome sequencing reads of its host *A. bruennichi*. The symbiont was first detected in *A. bruennichi* populations from Germany and Estonia *via* 16S rRNA gene amplicon sequencing (Sheffer et al. 2020). Phylogenetics revealed that the genome is not closely related to known *Mycoplasmatota* clade genomes. Detection of the symbiont in newly hatched spiderlings suggests vertical transmission to the offspring (Sheffer et al. 2020). Distinctive features include strong wasp spider host association, low GC content and a unique 16S rRNA gene sequence (Locus tag JTY60_02675; ncbi.nlm.nih.gov/datasets/gene/GCA_051094275.1/?gene_type = rRNA)

We therefore propose to treat this symbiont as a new candidate bacterial species, under the name *Candidatus* Argiopiplasma dusa.

Candidatus genus: ‘*Candidatus* Argiopiplasma’ (Ar.gi.o.pi.plas’ma. N.L. fem. n. Argiope, a spider genus; Gr. neut. n. plasma, anything formed or moulded, image, figure—referring to class Mycoplasmatales; N.L. neut. n. Argiopiplasma, a form from the Argiope spider genus)

*‘Candidatus* Argiopiplasma dusa’ (du’sa. N.L. fem. n.; dusa—name arbitrarily formed from a combination of letters to be treated as a noun in apposition“Dominant Unknown Symbiont of *A. bruennichi”* (DUSA)). We like to mention that the word **“dúša”** in Slavic languages mean spirit or soul. Type material is the genome assembly designated as CP070274.1 (NCBI GenBank) and GCF_051094275.1/GCA_051094275.1 (NCBI Genome) representing ‘*Candidatus* Argiopiplasma dusa’.

## Supplementary Material

fnag103_Supplemental_Files

## Data Availability

Whole genome *A. bruennichi* PacBio reads (Sequence Read Archive: SRR11652932) Whole genome *A. bruennichi* Illumina paired-end reads (European Nucleotide Archive: ERR574428) Assembly and annotation: accession CP070274 BioProject: PRJNA629526 BioSample: SAMN17496637 Assembly: GCF_051094275.1/GCA_051094275.1 Additional data is available from the corresponding author on request.
