# Supplementary material for: Genomic insights into Candidatus Argiopiplasma dusa, a bacterial symbiont of the wasp spider Argiope bruennichi
Source: FEMS Microbiol Lett. 2026 Sep 22;373:fnag103. doi: 10.1093/femsle/fnag103 (PMC13599016; doi:10.1093/femsle/fnag103)
Supplement: Supplementary file 1 [file fnag103_supplemental_files.zip › Fig-S2_SlowFast_Elife_markers_Endo_v3_win_EOL_S186_10perc.pdf]

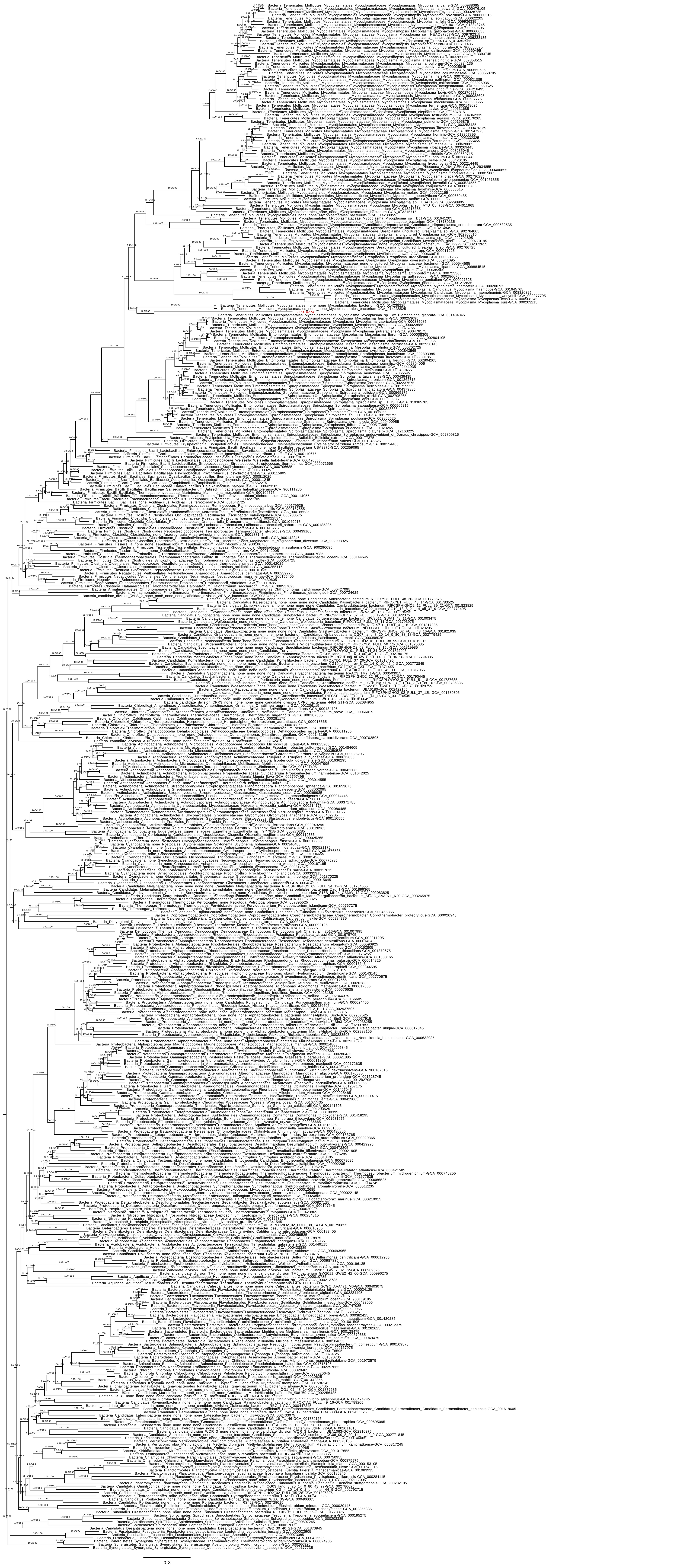
0.3
